# Differential Association of Gene Content Polymorphisms of Killer Cell Immunoglobulin-Like Receptors with Placental Malaria in HIV− and HIV+ Mothers

**DOI:** 10.1371/journal.pone.0038617

**Published:** 2012-06-08

**Authors:** Yusuf O. Omosun, Anna J. Blackstock, Wangeci Gatei, Allen Hightower, Anne Maria van Eijk, John Ayisi, Juliana Otieno, Renu B. Lal, Richard Steketee, Bernard Nahlen, Feiko O. ter Kuile, Laurence Slutsker, Ya Ping Shi

**Affiliations:** 1 Division of Parasitic Diseases and Malaria, Center for Global Health, Centers for Disease Control and Prevention, Atlanta, Georgia, United States of America; 2 Division of Global HIV/AIDS, Center for Global Health, Centers for Disease Control and Prevention, Atlanta, Georgia, United States of America; 3 Atlanta Research and Education Foundation, Atlanta, Georgia, United States of America; 4 Center for Vector Biology and Control Research, Kenyan Medical Research Institute, Kisumu, Kenya; 5 New Nyanza Provincial General Hospital, Ministry of Health, Kisumu, Kenya; 6 Child and Reproductive Health Group, Liverpool School of Tropical Medicine, Liverpool, United Kingdom; University of Alabama at Birmingham, United States of America

## Abstract

Pregnant women have abundant natural killer (NK) cells in their placenta, and NK cell function is regulated by polymorphisms of killer cell immunoglobulin-like receptors (KIRs). Previous studies report different roles of NK cells in the immune responses to placental malaria (PM) and human immunodeficiency virus (HIV-1) infections. Given these references, the aim of this study was to determine the association between KIR gene content polymorphism and PM infection in pregnant women of known HIV-1 status. Sixteen genes in the KIR family were analyzed in 688 pregnant Kenyan women. Gene content polymorphisms were assessed in relation to PM in HIV-1 negative and HIV-1 positive women, respectively. Results showed that in HIV-1 negative women, the presence of the individual genes KIR2DL1 and KIR2DL3 increased the odds of having PM, and the KIR2DL2/KIR2DL2 homozygotes were associated with protection from PM. However, the reverse relationship was observed in HIV-1 positive women, where the presence of individual KIR2DL3 was associated with protection from PM, and KIR2DL2/KIR2DL2 homozygotes increased the odds for susceptibility to PM. Further analysis of the HIV-1 positive women stratified by CD4 counts showed that this reverse association between KIR genes and PM remained only in the individuals with high CD4 cell counts but not in those with low CD4 cell counts. Collectively, these results suggest that inhibitory KIR2DL2 and KIR2DL3, which are alleles of the same locus, play a role in the inverse effects on PM and PM/HIV co-infection and the effect of KIR genes on PM in HIV positive women is dependent on high CD4 cell counts. In addition, analysis of linkage disequilibrium (LD) of the PM relevant KIR genes showed strong LD in women without PM regardless of their HIV status while LD was broken in those with PM, indicating possible selection pressure by malaria infection on the KIR genes.

## Introduction

In sub-Saharan Africa, it is estimated that 32 million pregnant women are at risk for *Plasmodium falciparum* infection annually [1]. The placenta, in particular, is highly susceptible to the infection and malaria parasite sequesters in vascular spaces, resulting in local inflammation [2]. Parity-dependent malarial immunity is acquired over consecutive pregnancies protecting multigravid women against the adverse effect of pregnancy-associated malaria, and as a consequence, in high transmission areas susceptibility to malaria decreases with increasing gravidity, whereas in areas of low endemicity, this trend is not as evident. The public health consequences of malaria in pregnancy also vary by transmission intensity: maternal illness, preterm births and fetal loss predominate in low transmission settings whereas maternal anemia, fetal growth restriction resulting in low birth weight and subsequent increased infant mortality are commonly seen in high transmission areas [3], [4], [5]. HIV-1 infection in women of reproductive age is another major public health problem and often coexists with malaria in pregnant women in sub-Saharan Africa [6]. HIV complicates the adverse consequences of placental malaria (PM) by increasing risk of infection, parasite densities and severity of the disease [7], [8] and by impairing the response to anti-malarial therapy [9]. On the other hand, malaria infection in pregnant women increases HIV-1 viral load [10], but the effect on perinatal mother to child transmission (MTCT) of HIV is not clear. Six studies addressed whether PM enhanced MTCT and all were conducted prior to the widespread introduction of antiretrovirals (ARVs) with conflicting conclusions [11], [12], [13], [14], [15], [16], showing increased MTCT in two studies in Uganda [11], [15], no effect in two other studies at the Kenya Coast [13], [14], and a significant protective effect in Mozambique and western Kenya [12], [16].

Pregnant women have abundant Natural Killer (NK) cells in the decidua of placenta. NK cells function by cell cytolysis or by cytokine production, and their functions have been implicated in the process of placentation and as first-line innate immune defense to infection during pregnancy [17]. Previous immunological studies have shown that increased IFN-γ expressing NK cells in the placenta were associated with a reduced risk of PM infection regardless of gravidity [18], and that HIV-1 infection could impair their ability to control PM, in part, by loss of IFN-γ production in placenta [19]. NK cells have also been associated with protection from HIV infection [20]; however high HIV viral loads cause expansion of functional deficient NK cells [21], [22]. These results suggest that NK cell related immunity is involved in both malaria infection during pregnancy and HIV infection, and malaria co-infection with HIV could have the potential to change NK cell mediated defense to both diseases.

Killer immunoglobulin (Ig)-like receptors (KIRs) are surface receptors expressed on NK cells, which modulate NK cell functions in response to changes caused by stress and infection [23]. The human KIR family comprises of 17 individual KIR genes located on chromosome 19q13.4, and nomenclature of the KIR genes is based on structural and functional characteristics [24]. A KIR with two extracellular Ig domains and short intracytoplasmic tail is named KIR2DS with a given number as a suffix, such as KIR2DS2 or KIR2SD4. A KIR with three extracellular Ig domains and long intracytoplasmic tail is named KIR3DL with a given number as a suffix, for example KIR3DL1 and KIR3DL2. Functionally, KIRs with short intracytoplasmic tails (S) activate NK cells and those with long intracytoplasmic tails (L) inhibit NK cells [24]. Some KIR genes have strong linkage disequilibrium (LD), and are thus usually inherited together [25]. KIRs are the second most polymorphic family in the mammalian genome after the major histocompatibility complex (MHC), and their polymorphisms differ between individuals mainly at three levels: 1) gene content polymorphism of KIR family; 2) allelic diversity of individual KIR genes; and 3) variation in binding specificity of individual KIR to HLA class I ligands [26]. This extreme polymorphism has made the KIR gene family a compelling genetic target for disease association studies, particularly for infectious diseases in which NK cells are involved [27], [28]. During pregnancy, NK cells in the decidua of human placenta mainly expresses inhibitory KIR2DL1, KIR2DL2, and KIR2DL3, dual functional (inhibitory/activating) KIR2DL4 and activating KIR2DS1 with skewed distribution of HLA-C ligand in the trophoblast [17], [29], [30]. The NK cell functions in the placenta are in part modulated by the balance of inhibitory and activating signals from KIRs. The NK cell functions predominantly mediated by cytokine production in the placenta regulate pregnancy outcomes while the NK cell functions presumably mediated by cytolysis and cytokine production are essential in the defense against infections [29], [30], [31]. Therefore, KIR gene locus profiles in the placenta are essentially shaped by a combination of pregnancy success and pathogen defense during pregnancy

Polymorphisms of KIRs have been studied in both non-infectious and infectious diseases [32]. In pregnancy, polymorphisms of KIR gene content coupled with variation of HLA ligands have been associated with recurrent miscarriages [33], [34] and predisposition to pre-eclampsia [35]. Break in strong LD between KIR genes has also been found in leukemia patients [36]. The outcomes of several viral infections such as HIV/AIDS, HCV and HBV have also been related with KIR gene content polymorphism and/or allele diversity along with HLA ligand variation [37], [38], [39], [40]. So far, few studies have addressed the role of KIR gene polymorphism in malaria. One such study involved analysis of KIR genotypes and *in vitro* NK cell response to malaria parasite in non-immune donors and reported a relationship between inhibitory KIR3DL2*002 allele and high NK cell IFN-γ response [41]. Another small-scale field-based study conducted in no-pregnant adults in Solomon Island (n = 77) found that malaria positive individuals had a high frequency of KIR3DL1/KIR3DS1 heterozygosity in combination with non-deleted KIR2DS4 allelic variant [42]. The results from these limited studies suggest that KIRs are important candidate genes in host resistance to malaria infection.

Given the abundance of NK cells in the placentas of pregnant women, the biological relevance of the different roles of NK cells in the hosts' immune responses to PM and HIV infections, the role of KIR polymorphism in regulating NK function and the involvement of KIRs in malaria infection in non-pregnant women, the aim of this study was to determine whether KIR gene content polymorphism was associated with protection or susceptibility to PM infection in pregnant women of known HIV-1 status. Here, we provide the first evidence that 1) the KIR gene content polymorphism is associated with PM infection, 2) this association is affected by the HIV status of the pregnant women, and 3) the effect of KIR gene content polymorphism on PM infection in the HIV positive women is dependent on high CD4 cell counts. In addition, analysis of linkage disequilibrium (LD) of the PM relevant KIR genes further suggested malaria infection has a selection pressure on the genes.

## Materials and Methods

### Ethics Statement

Written informed consent was obtained from women at time of enrollment. Study methods were approved by the Kenya Medical Research Institute Ethical Review Committee, Nairobi, Kenya and the Institutional Review Board of the Centers for Disease Control and Prevention, Atlanta, GA, USA.

### Study Site and Population

The present study was integrated into an epidemiologic investigation of the relationship between PM and MTCT of HIV-1 (the ‘VT project’) carried out in western Kenya between 1996 and 2001. Methods for the epidemiological study have been described in detail elsewhere [43]. Briefly, at the time of the study, the transmission of malaria was intense and perennial, and *P. falciparum* accounted for about 98% of malarial infections. The residents in this area are predominantly of Luo ethnic group. Women with singleton uncomplicated pregnancies of at least 32 weeks gestation were enrolled if they had no known underlying chronic illnesses. Information on reproductive history, socio-demographics, malaria treatment, and heath/clinical status was collected at enrollment and delivery respectively. Blood samples were collected at enrollment, delivery and 1 month postpartum for malaria diagnosis, HB level determination, HIV-1 diagnosis, HIV-1 viral load and/or CD4 counts. In the main VT study, enrollment priority was given to HIV-positive women, and within HIV-negative women enrollment priority was given to the women with PM. A total of 269 HIV-negative and 829 HIV-positive pregnant women were enrolled in the main VT study. For this nested genetic study, blood samples from 210 HIV-negative and 478 HIV-positive pregnant women were tested for KIR gene content polymorphisms. Sample selection for this nested genetic study was based on two criteria: 1) women who had the diagnostic results for both PM and HIV-1 and the information on treatment for malaria during the third trimester of pregnancy, and 2) availability of samples for DNA purification among these women.

### Laboratory Procedures

#### Malaria and anemia diagnosis

PM was assessed on blood samples obtained from a shallow incision on the maternal side of the placenta. Thick smears made from peripheral and placental blood were examined by microscopy. The number of asexual parasites/300 leukocytes was counted. Parasite density was estimated on the assumption of 8000 leukocytes/µl. The presence of malarial pigment in placental intervillous macrophages was also recorded. Peripheral blood hemoglobin concentrations (g/dl) were quantified by use of the HemoCue system (HemoCue).

#### HIV serology diagnosis, CD4 counts and viral load

A primary Serostrip HIV-1/2 (Saliva Diagnostic Systems) and a confirmatory Capillus HIV-1/2 test (Cambridge Diagnostics) were used to determine maternal HIV status. CD4 cell counts in HIV-positive women were determined by standard fluorescent-activated cell-sorting analysis on whole blood (FACScan, Becton Dickinson). Maternal HIV-1 load was measured by use of the Roche Amplicor HIV-1 monitor (test version 1.0; Roche Diagnostics).

#### KIR Genotyping

DNA was extracted from the blood samples using the QIAamp DNA blood mini kit (Qiagen). KIR genotyping for 16 KIR genes was carried out using KIRSSO genotyping test based on the manufacturer instructions (One Lambda Inc.). Briefly, three PCR products were hybridized with a panel of beads labeled with KIR specific probes and results were read for each bead in a separate session on the Luminex 200 IS (Luminex Corp.). Data was acquired using the IS v2.3 software (Luminex Corp.), and HLA Fusion Beta software (One Lambda Inc.) was used to determine the presence or absence of individual KIR genes. Five positive control DNA samples from the International Histocompatibility Working Group (IHWG) with different profiles of KIR gene content were used in all experiments. In addition, quality assurance was conducted using another gene-specific method, KIRSSP [44], and the results from this method matched with that using the KIRSSO commercial kit.

### Definitions

#### Clinical endpoints and definitions

Peripheral parasitemia was defined as any asexual blood-stage parasites seen by microscopy on a thick smear. Thick smears were deemed negative if viewing 100 high-power fields revealed no parasites. PM was similarly defined. Placentas with malarial pigment in the absence of parasitemia were classified as negative for PM. Anemia was defined as a hemoglobin concentration <11 g/dL and severe anemia as <7 g/dL. The malaria-transmission season comprised the months of April–June and October–December. The variable “season of third trimester” was defined as the women experienced with malaria transmission season during 3 months before delivery. Newborns were classified as “preterm” if delivery occurred before 37 weeks gestation. Low birth weight (<2500 g) of newborn was based on weight measured to the nearest 10 grams within 24 hours of birth. Gravidity of mothers was stratified into primi-, secundi-, and multigravid. Maternal HIV-positive status was determined on the basis of antibodies to HIV-1, detected by 2 rapid serological tests. Viral loads were categorized as <1,000, 1000–9999, and > = 10,000 copies/mL of plasma and CD4 cell counts were compared with cut-offs of 350 and 500 cells/mL.

#### Definitions for KIR gene content polymorphisms

KIR gene content polymorphisms used in this study were defined at four different levels to explore any possible association with multiple malaria related outcomes as well as placental malaria/HIV co-infection. The first level was the presence or absence of 16 individual KIR genes; 8 inhibiting, 6 activating and 2 pseudogenes [32]. Secondly, based on results of single KIR gene content polymorphism in relation to PM in this study, reported linkage disequilibrium, and segregation as alleles, two gene pairs KIR2DL2/KIR2DL3 and KIR2DS2/KIR2DL2 were used to further assess KIR gene content polymorphisms. The third level was the conventional classification of Haplotypes A and B. Haplotype A was defined based on the presence of 9 KIR genes, KIR3DL3, KIR2DL3, KIR2DL1, KIR2DP1, KIR3DP1, KIR2DL4, KIR3DL1, KIR2DS4 and KIR3DL2 only, while all remaining individuals were defined as Haplotype B [45], [46], [47]. Fourth, KIR were grouped as previously reported genotypes AA, AB and BB, where genotype AA included individuals with KIR3DL3, KIR2DL3, KIR2DL1, KIR2DP1, KIR3DP1, KIR2DL4, KIR3DL1, KIR2DS4, and KIR3DL2, genotype BB were individuals who did not have KIR2DL1, KIR2DL3, KIR3DL1 and KIR2DS4, and all the remaining individuals not in AA or BB were grouped as genotype AB [33]. Lastly, the more polymorphic haplotype B was further defined into subtypes based on three genes used above (KIR2DL2, KIR2DL3 and KIR2DS2) which show selection expression (either exclusively or simultaneously). This was to increase the resolution on haplotype B as previous studies show conflicting content polymorphisms in the gene combinations. Some reports show KIR2DL2/KIR2DL3 as alleles of the same locus, KIR2DS2 always paired with 2DL2, and 2DS2/2DL2 both negatively associated with 2DL3 [25], [44], [46], [48]. However, others report that the relationships among the three genes are not exclusive [45], [49], [50]. In this study, subtypes B were defined as: B1 is the presence of KIR2DL3 with absence of KIR2DS2 and KIR2DL2; B2 is the presence of KIR2DL2 with absence of KIR2DL3 regardless of KIR2DS2; B3 is the presence of all three KIRs; and B4 is the presence of KIR2DL3 and presence of either KIR2DL2 or KIR2DS2.

#### Linkage disequilibrium (LD)

Linkage disequilibrium (LD) is a measure of non-random association of alleles at two loci [51]. LD was used in this study to assess the genetic association between KIR gene pairs (KIR2DL1, KIR2DL2, KIR2DL3, and KIR2DS2) relevant to PM in HIV-1 negative and positive pregnant women respectively. The computation of the LD [52] is based on the presence and absence of these PM-related genes. Positive LD is defined as when a gene pair is either present or absent together, while negative LD is when either one of the gene pair is present in the absence of the other.

### Statistical Analysis

Because of the stratified enrollment in the main epidemiological study, all analyses were stratified a priori by the mother's HIV status. PM was the primary outcome of interest. Parasite density (log transformed), anemia in the 3^rd^ trimester, preterm delivery, and low birth weight were secondary outcomes. Associations between mother and infant characteristics and the primary outcome variable PM were first examined using the Pearson exact chi-square test for the categorical variables and the Wilcoxon rank sum test for continuous variables.

The associations between KIR gene content polymorphisms and PM were examined using univariable and multivariable logistic regression models. KIR categories with the highest frequencies were used as reference groups for analyses involving gene pairs, genotypes, haplotypes, and subtypes B. For individual genes, the reference groups consisted of women without the specific gene of interest. The final models controlled for gravidity, antimalarial use during the third trimester, and predominant third trimester season based on their significance in preliminary analyses as well as to biological plausibility and prior knowledge. Similar analyses were performed for the secondary outcomes parasite density, anemia, pre-term delivery, and low birth weight. Multivariable analysis of variance (ANOVA) was used for the log transformed parasite density outcome, controlling for the variables mentioned above.

In HIV-1 positive women, the effects of CD4 cell counts and viral load on the association between KIR gene content polymorphisms and PM were assessed by allowing for interaction between KIR gene content polymorphisms and CD4 cell counts and viral load groups. Women with <350 cells/µl were classified as having low CD4 cell counts, and women with ≥10,000 copies/ml were classified as having high viral load. CD4 cell count and viral load interactions were included in separate models, and multivariable models controlled for the factors listed above.

The method of Benjamini and Hochberg [53] was used to correct for multiple comparisons in the regression analysis by controlling the false discovery rate (FDR) and the cut-off was set at 0.05. The FDR was controlled separately for three groups of models: single gene models, gene pair models, and models for haplotype, genotype, and subtype B. Within each group, the FDR was controlled independently for models for HIV-1 positive and HIV-1 negative mothers. Uncorrected P-values are given in all tables, with “*” denoting those tests which were significant (at α = 0.05) before correcting for multiple comparisons but not after, and with “**” denoting those tests that remained significant after multiple comparison adjustments. Analyses were performed using SAS version 9.2.

Arlequin was used to calculate the LD values (*D*, and *D*′) between four PM-relevant KIR genes (KIR2DL1, KIR2DL2, KIR2DL3 and KIR2DS2) using pair-wise test based on the presence or absence of the respective genes [52]. The pair-wise test was not possible to conduct if one gene is monomorphic (all presence or all absence among individuals). LD classical coefficient (*D*) measuring deviation from random association between the gene pairs was expressed as *D = Pij- Pi Pj*, where *Pi* and *Pj* are gene frequencies at loci *i* and *j*. Taking into consideration the role of gene frequencies in the classical LD coefficient *D*, a *D′* was given by standardizing *D* with the maximum value it can have (*D*′ = *D*/*D*_max_) [54]. The statistical significance of both positive and negative LD values was assessed by Fisher exact test, and corrected using Bonferroni adjustment, with LD being significant at p<0.002.

## Results

### Characteristics of women by PM status

The clinical and demographic characteristics of the study participants are shown in Table 1. In HIV-1 negative women, PM infection was significantly associated with younger maternal age, primigravidity, transmission season, and semi urban residence. Similarly, young age and first pregnancy were risk factors for PM among HIV-1 positive individuals. A history of antimalarial treatment in the third trimester was protective against PM among HIV-1 positive women, but not among HIV-1 negative women. Among HIV-1 positive women, there was no significant difference in the maternal CD4 cell counts and HIV-1 load between PM+ and PM− mothers. Placental malaria was associated with maternal peripheral malaria, malaria pigment in the placenta, and a higher risk of delivering low birth weight newborns.

**Table 1:** Characteristics of HIV-1 negative and HIV-1 positive women, by placental malaria (PM) status.

	HIV-1 negative women			HIV-1 positive women		
Category/Characteristics	PM+ (N = 116)	PM− (N = 93)^a^	P	PM+ (N = 105)^a^	PM− (N = 374)	P
	n (%)^a^	n (%)		n (%)	n (%)^a^	
Maternal peripheral malaria						
Present during third trimester	32/98 (32.7)	9/75 (12.0)	0.002	41/83 (49.4)	36/312 (11.5)	<0.001
Present at delivery	80/113 (70.8)	5/92 (5.4)	<0.001	67/103 (65.1)	13/363 (3.6)	<0.001
Preterm delivery, <37 weeks	9 (7.8)	6 (6.5)	0.79	12 (11.4)	26/373 (7.0)	0.15
Newborn birth weight, <2500 g	9 (7.8)	0 (0)	0.005	11 (10.5)	19 (5.1)	0.065
Maternal anemia at third trimester, g/dl						
<11	77/98 (78.6)	64/75 (85.3)	0.32	62/82 (75.6)	269/317 (84.9)	0.050
<7	10/98 (10.2)	6/75 (8.0)	0.79	7/82 (8.5)	22/317 (6.9)	0.63
Maternal anemia at delivery, g/dl						
<11	74/111 (66.7)	48/90 (53.3)	0.060	67/102 (65.7)	206/361 (57.1)	0.14
<7	9/111 (8.1)	3/90 (3.3)	0.23	12/102 (11.8)	23/361 (6.4)	0.088
Malaria pigment	103 (88.8)	2 (2.2)	<0.001	91 (86.7)	0 (0)	<0.001
Mother's age, mean years ± SD	19.5±3.9	22.5±4.7	<0.001	22.0±4.8	22.7±4.3	0.016
Gravidity						
Primigravid	77 (66.4)	31 (33.3)	<0.001	47 (44.8)	115 (30.8)	0.015
Secundigravid	17 (14.7)	31 (33.3)	……	21 (20.0)	116 (31.0)	……
Multigravid	22 (19.0)	31 (33.3)	……	37 (35.2)	143 (38.2)	……
Antimalarial use, treated during third trimester	27/115 (23.5)	29 (31.2)	0.27	23/104 (22.1)	130/372 (35.0)	0.017
Place of living, semi-urban (vs. urban)	42 (36.2)	21/92 (22.8)	0.048	28/104 (26.9)	94/372 (25.3)	0.80
Predominant season of third trimester, Malaria transmission season	86 (74.1)	56 (60.2)	0.037	61 (58.1)	208 (55.6)	0.66
Maternal CD4 cell count, cells/ul						
<350	……	……	……	17/93 (18.3)	63/333 (18.9)	1.00
<500	……	……	……	37/93 (39.8)	127/333 (38.1)	0.81
Median (interquartile range)^b^	……	……	……	589 (419–714)	563 (379–818)	0.71
Maternal HIV-1 load, copies/ml						
<1000	……	……	……	36/79 (45.6)	151/299 (50.5)	0.33
1000–9999	……	……	……	24/79 (30.4)	98/299 (32.8)	……
≥10,000	……	……	……	19/79 (24.1)	50/299 (16.7)	……
Median (interquartile range)^c^	……	……	……	1338 (200–9039)	982 (200–5145)	0.26

Note: Shown is percentage of women in given PM and HIV category with specified characteristic unless otherwise noted. P-values are based on Pearson chi-square test or Wilcoxon test. PM+ = placental malaria present, PM− = placental malaria absent.

a If N differs from number listed, n/N is given.

b N = 94 for PM+ and N = 335 for PM−.

c N = 79 for PM+ and N = 299 for PM−.

### Association between KIR gene content polymorphisms and PM infection in HIV-1 negative and HIV-1 positive women

The first analysis was to test if any individual KIR gene was associated with PM (Table 2). The framework genes KIR3DL2 and KIR3DL3 were present in all samples analyzed and subsequently excluded from the association analysis. In addition, the two pseudogenes, KIR2DP1 and KIR3DP1, were also excluded from the association analysis. Among HIV-1 negative women, individuals with KIR2DL1 and KIR2DL3 were more likely to have PM (OR = 15.45, 95% CI = >2.05, p = 0.006**, and OR = 5.03, 95% CI = 1.63–15.54, p = 0.005** respectively). In contrast, in HIV-1 positive women, the presence of only KIR2DL3, but not KIR2DL1, was associated with protection from PM (OR = 0.41, 95% CI = 0.24–0.72, p = 0.002**).

**Table 2:** Adjusted Odds ratios for individual KIR genes and KIR gene combinations in HIV-1 negative and HIV-1 positive women, outcome placental malaria (PM) status.

	HIV-1 negative women				HIV-1 positive women			
	Frequencies				Frequencies			
	PM+ (N = 116)	PM− (N = 93)	Adjusted OR	P	PM+ (N = 105)	PM− (N = 374)	Adjusted OR	P
	n (%)	n (%)	(95% CI)		n (%)	n (%)	(95%CI)	
Individual KIR Genes								
KIR2DL1	116 (100)	87 (93.6)	15.45(>2.05)^a^	0.006**	101 (96.2)	364 (97.3)	0.69 (0.21–2.30)	0.55
KIR2DL2	57 (49.1)	53 (57.0)	0.81 (0.44–1.49)	0.50	61 (58.1)	192 (51.3)	1.23 (0.78–1.92)	0.37
KIR2DL3	111 (95.7)	78 (83.9)	5.03 (1.63–15.54)	0.005**	79 (75.2)	328 (87.7)	0.41 (0.24–0.72)	0.002**
KIR2DL4	116 (100)	92 (98.9)	0.49 (>0.01)^a^	1.00	104 (99.1)	372 (99.5)	0.43 (0.04–5.14)	0.51
KIR2DL5	60 (51.7)	57 (61.3)	0.72 (0.39–1.32)	0.29	66 (62.9)	205 (54.8)	1.38 (0.87–2.18)	0.17
KIR2DS1	25 (21.6)	19 (20.4)	1.27 (0.61–2.64)	0.52	15 (14.3)	84 (22.5)	0.59 (0.32–1.08)	0.086
KIR2DS2	46 (39.7)	46 (49.5)	0.75 (0.41–1.36)	0.34	52 (49.5)	161 (43.1)	1.24 (0.80–1.93)	0.34
KIR2DS3	25 (21.6)	23 (24.7)	0.78 (0.39–1.58)	0.49	24 (22.9)	66 (17.7)	1.45 (0.85–2.48)	0.18
KIR2DS4	112 (96.6)	85 (91.4)	2.71 (0.73–10.11)	0.14	100 (95.2)	364 (97.3)	0.52 (0.17–1.60)	0.25
KIR2DS5	43 (37.1)	37 (39.8)	0.99 (0.54–1.83)	0.98	49 (46.7)	167 (44.7)	1.06 (0.68–1.66)	0.79
KIR3DL1	113 (97.4)	89 (95.7)	1.45 (0.29–7.30)	0.65	104 (99.1)	369 (98.7)	1.42 (0.16–12.61)	0.75
KIR3DS1	19 (16.4)	10 (10.8)	1.77 (0.73–4.33)	0.21	9 (8.6)	50 (13.4)	0.63 (0.30–1.35)	0.23
KIR Gene Combinations								
KIR2DL2/KIR2DL3^c^								
2DL2/2DL3	52 (44.8)	38 (40.9)	1.10 (0.58–2.11)	0.77	35 (33.3)	147 (39.4)	0.88 (0.53–1.46)	0.61
2DL2/2DL2	5 (4.3)	15 (16.1)	0.21 (0.07–0.67)	0.008**	26 (24.8)	45 (12.1)	2.37 (1.30–4.30)	0.005**
2DL3/2DL3	59 (50.9)	40 (43.0)	1	……^b^	44 (41.9)	181 (48.5)	1	……^b^
KIR2DS2/KIR2DL2								
−/−	58 (50.0)	38 (40.9)	1	……^b^	43 (41.0)	176 (47.1)	1	……^b^
+/+	45 (38.8)	44 (47.3)	0.75 (0.40,–1.43)	0.86	51 (28.6)	155 (32.4)	0.95 (0.57–1.61)	0.86
+/−	1 (0.9)	2 (2.2)	0.91 (0.08–11.04)	0.94	1 (1.0)	6 (1.6)	0.64 (0.07–5.63)	0.69
−/+	12 (10.3)	9 (9.7)	1.09(0.39–3.04)	0.87	10 (9.5)	37 (9.9)	1.00 (0.45–2.22)	1.00

Note: PM+ = Placental malaria present, PM− = Placental malaria absent. OR = Odds ratio, CI = Confidence interval. P-values are derived from multivariable logistic regression, controlling for gravidity, anti-malarial use during third trimester, and malaria transmission season. Due to missing covariates in models, 115 PM+ and 93 PM− used for HIV-1 negative; 104 PM+ and 372 PM− used for HIV-1 positive. False Discovery Rate (FDR) was used to correct P-values.

‘*’ signifies uncorrected P-value that is <0.05 but is non-significant after correction.

‘**’ signifies uncorrected P-value that is significant after correction.

a Derived using exact logistic regression, median unbiased estimate reported.

b Reference group was selected based on the group with highest frequency.

c For HIV-1+ women, 373 women were PM−.

Secondly, based on the above key finding of the individual gene KIR2DL3, its segregation as an allele with KIR2DL2, and the close relationship between KIR2DL2 and KIR2DS2, two KIR gene pairs, KIR2DL2/KIR2DL3 and KIR2DS2/KIR2DL2, were assessed in relation to PM (Table 2). For the first gene pair, the highest proportions of women were either KIR2DL2/KIR2DL3 heterozygotes or KIR2DL3/KIR2DL3 homozygotes while KIR2DL2/KIR2DL2 homozygotes comprised a lower proportion in both HIV-1 positive and negative women (Table 2). In HIV-1 negative women, the KIR2DL2/KIR2DL2 homozygotes were less likely to have PM (OR = 0.21, 95% CI = 0.07–0.67, p = 0.008**). In contrast, the reverse relationship was observed in HIV-1 positive women, with these individuals being more likely to have PM (OR = 2.37, 95% CI = 1.30–4.30, p = 0.005**). For the second gene pair, KIR2DS2/KIR2DL2, majority of women either had both genes present or absent together (Table 2). A smaller proportion (approximately 10%) had KIR2DL2 present and KIR2DS2 absent while the least had only KIR2DS2 in the absence of KIR2DL2. No association was found between the KIR2DS2/KIR2DL2 pair and PM in this study.

Lastly, the relationships between KIR haplotypes, subtypes B, or genotypes and PM were examined (Table 3). There was no association of KIR haplotypes A and B with PM in both HIV-1 negative and HIV-1 positive women; however, an association between subtype B2 and PM was observed. A large proportion of subtype B2 comprised of KIR2DL2/KIR2DL2 homozygous (90%) in the presence of KIR2DS2 while approximately 10% had only KIR2DL2/KIR2DL2 and the absence of KIR2DS2. In HIV-1 negative women, individuals with subtype B2 were less likely to have PM (OR = 0.19, 95% CI = 0.06–0.62, p = 0.006**). In contrast, among HIV-1 positive women, subtype B2 was associated with increased susceptibility to PM (OR = 2.18, 95% CI = 1.17–4.06, p = 0.014*) (Table 3), although this was not statistically significant after correcting for multiple comparisons. This subtype B2 result further confirmed that KIR2DL2/2DL2 homozygous observed in the gene pair comparison (Table 2), regardless of KIR2DS2, plays a major role in PM and PM/HIV co-infection. Interestingly, while no association with PM was found for subtype B3 (KIR2DL2/KIR2DL3 and KIR2DS2 present together), this subtype B3 group comprised almost a third of all the individuals in this study (Table 3), an unusual finding when considering previous reports that showed complete negative or positive association for the three genes. No association was found between KIR genotypes AA, AB and BB and PM in this study (Table 3).

**Table 3:** Adjusted Odds ratios for KIR haplotypes, B subtypes and KIR genotypes in HIV-1 negative and HIV-1 positive women, outcome placental malaria (PM) status.

	HIV-1 negative women				HIV-1- positive women			
	Frequencies				Frequencies			
	PM+ (N = 116)	PM− (N = 93)	Adjusted OR	P	PM+ (N = 105)	PM− (n = 374)	Adjusted OR	P
	n (%)	n (%)	(95% CI)		n (%)	n (%)	(95%CI)	
Haplotypes								
A	49 (42.2)	30 (32.3)	1.39 (0.74–2.59)	0.30	36 (34.3)	134 (35.8)	0.97 (0.61–1.54)	0.88
B	67 (57.8)	63 (67.7)	1	……^b^	69 (65.7)	240 (64.2)	1	……^b^
B Subtypes								
A	49 (42.2)	30 (32.3)	1	……^b^	36 (34.3)	134 (35.8)	1	……^b^
B1	12 (10.3)	8 (8.6)	0.86 (0.29–2.58)	0.79	9 (8.6)	46 (12.3)	0.80 (0.35–1.82)	0.60
B2	5 (4.3)	15 (16.1)	0.19 (0.06–0.62)	0.006**	26 (24.8)	45 (12.0)	2.18 (1.17–4.06)	0.014*
B3	40 (34.5)	29 (31.2)	0.99 (0.48–2.07)	0.98	29 (27.6)	115 (30.8)	0.86 (0.49–1.50)	0.59
B4	10 (8.6)	11 (11.8)	0.78 (0.28–2.22)	0.64	5 (4.8)	34 (9.1)	0.48 (0.17–1.35)	0.16
Genotypes								
AA	46 (39.7)	30 (32.3)	1.17 (0.60–2.30)^a^	0.73	34 (32.4)	129 (34.5)	0.94 (0.59–1.51)	0.81
AB	70 (60.3)	61 (65.6)	1	……^b^	70 (66.7)	243 (65.0)	1	……^b^
BB	0 (0)	2 (2.2)	0.29 (0–4.50)^a^	0.37	1 (1.0)	2 (0.5)	2.28 (0.19–27.29)	0.52

Note: PM+ = Placental malaria present, PM− = Placental malaria absent. OR = Odds ratio, CI = Confidence interval. P-values are derived from multivariable logistic regression, controlling for gravidity, anti-malarial use during third trimester, and malaria transmission season. Due to missing covariates in models, 115 PM+ and 93 PM− used for HIV-1 negative; 104 PM+ and 372 PM− used for HIV-1 positive. False Discovery Rate (FDR) was used to correct P-values.

‘*’ signifies uncorrected P-value that is <0.05 but is non-significant after correction.

‘**’ signifies uncorrected P-value that is significant after correction.

a Derived using exact logistic regression, median unbiased estimate reported.

b Reference group was selected based on the group with highest frequency.

### Effect of CD4 counts and HIV-1 load on the relationship between KIR gene content polymorphism and PM infection in HIV-1 positive women

Since CD4 cell counts and HIV-1 viral load are important determinants of HIV progression, we further explored whether they affected the observed relationship between KIR gene content polymorphisms (individual KIR2DL3 and KIR2DL2/KIR2DL2 homozygote) and PM. The interaction terms of KIR2DL3 and KIR2DL2/KIR2DL2 with CD4 cell counts (<350 or ≥350) and viral load (<10,000 or ≥10,000) were not significant but were retained in models in order to produce individual odds ratios for CD4 and viral load categories. Associations were evident in women with CD4 cell counts ≥350 cells/µl. The presence of KIR2DL3 alone was associated with protection from PM and KIR2DL2/KIR2DL2 homozygotes rendered women more susceptible to PM. These associations remained statistically significant after correcting for multiple comparisons (Table 4). However, none of these associations were seen in women with CD4 cell counts <350 cells/µl. Among women with lower viral load, KIR2DL3 was associated with a decreased susceptibility to PM. The association was not significant after correcting for multiple comparisons and was not detected in women with higher viral load (Table 4).

**Table 4:** Effect of CD4 cell count and HIV viral load on the relationship of KIR2DL3 and KIR2DL2/KIR2DL2 with PM in HIV-1 infected women, outcome placental malaria (PM) status.

Parameter, level	No. of women	PM (%)	Adjusted OR	P^a^
	(No. with Gene)		(95%CI)	
**KIR2DL3**			**Present vs. Absent**	
CD4 cell count, cells/µl				
<350	80 (69)	21.3	0.70 (0.16–3.03)	0.63
≥350	346 (296)	22.0	0.35 (0.18–0.67)	0.002**
HIV-1 load, copies/ml				
<10,000	309 (267)	19.4	0.44 (0.21–0.93)	0.031*
≥10,000	69 (57)	27.5	1.20 (0.28–5.24)	0.80
**KIR2DL2/KIR2DL2**			**2DL2/2DL2 vs. 2DL3/2DL3**	
CD4 cell count, cells/µl				
<350	80 (11)	21.3	1.13 (0.23–5.44)	0.88
≥350	345(49)	22.0	2.82 (1.40–5.68)	0.004**
HIV-1 load, copies/ml				
<10,000	309 (42)	19.4	2.10 (0.95–4.68)	0.069
≥10,000	69 (12)	27.5	1.13 (0.24–5.40)	0.88

Note: PM = Placental malaria. OR = Odds ratio, CI = Confidence interval. P*^a^* derived from multivariable logistic regression, controlling for gravidity, anti-malarial use during third trimester, and malaria transmission season. Similar to previous analysis the reference group was selected based on the group with highest frequency for the significant gene pair and based on the absence for significant individual gene.

False Discovery Rate (FDR) was used to correct P-values.

‘*’ signifies uncorrected P-value that is <0.05 but is non-significant after correction.

‘**’ signifies uncorrected P-value that is significant after correction.

### KIR genes LD and PM infection among HIV-1 negative and HIV-1 positive women

Malaria has a strong selective pressure on human genes [55]. In order to further evaluate the relationship between the KIR genes and PM, the LDs for PM-relevant KIR2DL1, KIR2DL2, KIR2DL3, and KIR2DS2 were calculated in HIV-1 positive and negative mothers stratified by PM status (Table 5). Among women who were HIV-1 positive, the gene pairs KIR2DL1/KIR2DL3 and KIR2DL2/KIR2DS2 were in significant positive LD, while KIR2DL2/KIR2DL3 and KIR2DL3/KIR2DS2 were in significant negative LD for both women with or without PM. In contrast, among HIV-1 negative women where LD test was possible, only KIR2DL2/KIR2DS2 gene pair was in significant positive LD in women with or without PM. In addition, distinct differences were observed in LD for some of KIR gene pairs between PM+ and PM- women regardless of the HIV status. The gene pairs KIR2DL1/KIR2DL2 and KIR2DL1/KIR2DS2 (HIV-1 positive), KIR2DL2/KIR2DL3 and KIR2DL3/KIR2DS2 (HIV-1 negative) were in significant negative LD in women without PM whereas LD was insignificant in all the respective pairs among women with PM. The significant negative LDs among the women without PM is similar to that observed in a previous study among the general African population [49], while the break in LD among the women with PM is unusual.

**Table 5:** Linkage disequilibrium (LD) for pairs of KIR genes in HIV-1 negative and HIV-1 positive women, by placental malaria (PM) Status.

		KIR2DL2					KIR2DL3					KIR2DS2				
		HIV+		HIV−		African*	HIV+		HIV−		African*	HIV+		HIV		African*
		PM−	PM+	PM−	PM+		PM−	PM+	PM−	PM+		PM−	PM+	PM−	PM+	
**KIR2DL1**	D	−0.0130	−0.0160	−0.0280	–	−0.1900	0.0180	0.0290	0.0540	–	0.2100	−0.0150	−0.0100	−0.0330	–	−0.3400
	D′	−1.0000	−1.0000	−1.0000	–		0.7720	1.0000	1.0000	–		−1.0000	−0.5050	−1.0000	–	
	*p*	0.0020	0.0830	0.0280	–	<0.0001	0.0001	0.0004	0.0001	–	<0.0001	0.0002	0.2990	0.0110	–	<0.0001
**KIR2DL2**	D						−0.0570	−0.1040	−0.0690	−0.0220	−0.2700	0.1930	0.1980	0.1910	0.1930	0.1600
	D′						−0.9550	−1.0000	−1.0000	−1.0000		0.9230	0.9540	0.8990	0.9570	
	*p*						0.0001	0.0001	0.0001	0.0200	<0.0001	0.0001	0.0001	0.0001	0.0001	0.0100
**KIR2DL3**	D											−0.0540	−0.0870	−0.0820	−0.0260	−0.2800
	D′											−0.7710	−0.6950	−1.0000	−1.0000	
	*p*											0.0001	0.0001	0.0001	0.0050	<0.0001

Classical coefficient D measures deviation from random association between the gene pairs, and D′ is given by *D* with the maximum value it can have [54]. ‘–’ denotes D value not determined due to monomorphism in one gene. LD was significant at P<0.002. African* is linkage disequilibrium cited from a previous population study [49].

### Association between KIR gene content polymorphisms and PM secondary outcomes in HIV-1 negative and HIV-1 positive women

There was no association between the KIR gene content polymorphisms with any of the PM secondary outcomes in HIV-1 positive women. However, in HIV-1 negative women maternal anemia was associated with KIR gene content polymorphisms in which women with subtype B1 (KIR2DS2−, KIR2DL2−, and KIR2DL3+) were less likely to have anemia and the association remained statistically significant after correction for multiple comparison (data not shown).

## Discussion

In this study, we identified associations between KIR2DL1, KIR2DL2, and KIR2DL3 genes and PM infection. The data shows that in HIV-1 negative women, the presence of the individual genes KIR2DL1 and KIR2DL3 increased the odds for having PM. Further analysis of this group showed that KIR2DL2/KIR2DL2 homozygotes were less likely to have PM (Tables 2 & 3). In contrast, amongst HIV-1 positive women, the presence of individual KIR2DL3, but not individual KIR2DL1, was associated with protection from PM and the women with KIR2DL2/KIR2DL2 homozygote were more likely to have PM (Tables 2 & 3). Furthermore, when the HIV-1 positive mothers were stratified by CD4 counts, the reverse associations between KIR gene polymorphism and PM remained only in the individuals with high CD4 cell counts, but not in those with low CD4 cell counts. Collectively, the results suggest that inhibitory KIR2DL2 and KIR2DL3, which are alleles of the same locus, play a role in the inverse effects on PM and PM/HIV co-infection, and the effect of KIR gene content polymorphisms on PM in HIV-1 women is dependent on high CD4 counts.

The fundamental differences in KIRs and their ligand interaction between malaria and HIV infections and between placental compartments and peripheral blood may explain the results from the current genetic association study. Numerous studies on HIV-1 infection have pointed out the role of the interactions between inhibitory KIR genes and HLA class I ligands in the susceptibility to HIV infection. However, considering the erythrocytes, host cells for malaria parasites, are deficient in expression of HLA class I molecules, it is possible that novel ligands are involved in KIR's recognition and regulatory role in PM infection. It has been shown that malaria parasite infected erythrocytes (IEs) bind directly to NK cells, by attaching *P. falciparum* erythrocyte membrane protein-1 (pfEMP1) on IEs acting as a ligand to chondroitin sulfate A (CSA) on the NK cells [56]; however the binding of IEs on its own does not activate the NK cells [56]. Other studies reported that early NK cell derived IFN-γ response is absolutely dependent upon the contact with IEs and the cytokine production by accessory cells (monocytes and myeloid dendritic cells) [57]. Importantly, this ability of NK cells to react to the accessory cell-derived cytokines is dictated by the KIR genotype [58]. Taken together, this explains that the KIR without HLA class I ligand could operate independently, leading to protection (KIR2DL2) against or susceptibility (KIR2DL3 and KIR2DL1) to malaria infection in HIV-1 negative women, possibly by the binding of pfEMP1 on IEs to CSA on NK cells and the cross talking between accessory cells and NK cells. Alternatively, it is also possible that placental compartment has a unique KIR-ligand recognition. Sequestration of malaria parasites in the placenta is mediated by the cytoadherence of pfEMP1 on IEs to CSA expressed on placental syncytiotrophoblast [2]. PfEMP1 is known to be highly diverse and a recent study conducted in Kenya reported significant sequence variations in the placental parasite specific pfEMP1 [59], [60]. It is also known that placental trophoblast is skewed towards the production of HLA-C, the ligand for KIR2DL1, KIR2DL2 and KIR2DL3 expressed on decidual NK cells. We, therefore, speculate that the cytoadherence of the polymorphic placental malaria specific pfEMP1 to trophoblasts could regulate HLA-C expression level in the placenta according to the specific variation of pfEMP1, thus affecting the binding of HLA-C to KIR2DL2 or KIR2DL3 and KIR2DL1 in different ways. However, this hypothesis while plausible needs to be tested. A previous report showed that placental malaria infection decreased expression of HLA-G, a non-classical MHC class I molecule, in trophoblast [61].

HIV status appeared to modify the effect of KIRs on PM, reversing the association between the same set of KIR genes and PM infection. Although the underlying functional mechanisms for this switch remains unclear, broadly it is likely that a combination of HIV-specific KIRs and their ligand interactions and other non-genetic factors due to HIV-1 infection may drive the inversion of the association between KIR gene content polymorphisms and PM infection. Previous studies reported that gene content polymorphism of KIR2DL2 and KIR2DL3 in relation to HLA-C ligand controls the susceptibility to HIV infection in sex workers and in perinatal transmission model [38], [62]. It is possible that the interactions of KIR2DL2/2DL3 with its ligands which are specific for HIV may modify the relationship between KIR and PM and that this modification could be mediated by quantitative alteration of cellular expression of HLA class I ligands on T cells by HIV virus, thus reversing the association between KIR2DL2/KIR2DL3 and PM infection in HIV-1 positive women. This is supported by the studies that have described the selective down-regulation of HLA class I ligands (including HLA-C) expression on T cells by HIV virus [63]. Interestingly, another earlier study reported an induced expression of HLA class I molecules on erythrocytes in patients with HIV infection due to the effect of increased endogenous production of interferon-α [64]; thus supporting a possible interaction between the induced HLA class I molecules on infected erythrocytes and KIR on NK cells in malaria co-infection with HIV; however, this hypothesis needs to be further tested.

In HIV infection, depletion and functional impairment of CD4+ T cells is a key marker indicating progression to immuno-compromised status, where the host could be rendered more susceptible to malaria, tuberculosis and other opportunistic infections [65], [66], [67]. Thus, HIV infected individuals with high CD4 counts presumably have a better general immune capacity including the immune regulatory functions compared to those with low CD4 counts. In the present study, when we further stratified HIV-1 positive women by CD4 counts we observed the association of the KIR gene polymorphism with PM infection only in the individuals with high CD4 cell counts but not those with low CD4 counts (Table 4). This result could be expected when considering CD4 T cells as a marker for general immune status and KIRs as an immune regulator. HIV positive women with high CD4 counts maintain their general immune capacity including KIR regulatory role; as such, the undamaged KIR regulatory function in these women with different KIR genetic backgrounds could then regulate immune effectors (such as NK cells) that further determine their susceptibility to PM infection. In contrast, the women with low CD4 counts, even with different KIR genetic backgrounds, could lose their general immune capacity including KIR regulatory function; thus no association between KIR gene polymorphisms and PM could be predicted in this group. Although the immune functional pathways involved in this process are still unknown, a previous study has shown that there is a difference in HLA class I alleles expression, ligands for KIRs, between individuals with low and high CD4+ T cell counts [68].

In this study, we also further conducted the pair-wise LD analysis for PM-relevant KIR2DL1, KIR2DL2 and KIR2DL3 as well as KIR2DS2 and assessed the associations in the haplotype B subtypes. Four gene pairs (KIR2DL1/KIR2DL3, KIR2DL2/KIR2DS2, KIR2DL2/KIR2DL3 and KIR2DL3/KIR2DS2) were in significant LD in HIV-1 positive women regardless of PM status whereas only one gene pair KIR2DL2/KIR2DS2 was in significant LD in HIV-1 negative women. Most importantly, we further found that the four gene pairs, KIR2DL1/KIR2DL2 and KIR2DL1/KIR2DS2 (HIV-1 positive) and KIR2DL2/KIR2DL3 and KIR2DL3/KIR2DS2 (HIV-1 negative), were in significant LDs in the women without PM, consistent with LDs in the general African populations [49]. However, the findings of the disruption of LDs for these four respective pairs in the women with PM and the high proportion of women with all three 2DL3, 2DL2 and 2DS2 present together (subtype B3) in the study population, are unusual. Previous studies report the KIR2DL2/KIR2DL3 pair is usually in complete negative LD while KIR2DS2, where present, always pairs with KIR2DL2 [25], [46], [48]. In contrast, other studies show the occurrence of the three gene pairs together (with no report of disease association) [49], [69], [70], or break of LD associated with disease [36]. Gene pairs showing strong LD indicate non-random association at the population level and the observed disruption in LDs associated with PM in this study suggests possible selection by malaria infection. It has been suggested that KIR are probably under strong selective pressure due to the influence of pathogens [27] and many studies show that malaria has a strong selection pressure on human genes [55]. In addition, the disruption of LDs for different gene pairs could also partly explain the opposite associations between KIR and PM in HIV-1 negative and positive women.

Lastly, the association between the secondary outcomes (parasite density, anemia, pre-term delivery, and low birth weight) and KIR gene content polymorphisms was also analyzed. We only observed that HIV-1 negative women with subtype B1 (KIR2DS2-, KIR2DL2, and KIR2DL3+) were less likely to be anemic (data not shown). At the moment, the actual mechanism by which subtype B1 would regulate anemia in pregnant women is still unknown. However, the genotype AA which is the opposite to B1 subtype has been linked to predisposition to preeclampsia and recurrent miscarriages [34], [35], and there is an overlap of symptoms between placental malaria and preeclampsia [71]. Thus, KIR might play a role in regulating these symptoms, one of which is anemia.

To our knowledge, this is the first study to demonstrate an association of KIR gene content polymorphisms with PM infection in pregnant women with or without HIV-1 co-infection. Our study is complex and makes use of mother samples from a PM/HIV interaction epidemiological study with good clinical data. The results suggest that inhibitory KIR2DL2 and KIR2DL3, which are alleles of the same locus, play a role in the inverse effects on PM alone and PM/HIV co-infection and the effect of KIR gene content polymorphisms on PM in HIV-1 positive women is dependent on high CD4 cell counts. In addition, the results from our study suggest that anemia during pregnancy might be regulated by KIR genes, and malaria infection could have a selective pressure on KIR2DL2, KIR2DL3 and other relevant KIR genes. Our results stress the need to further type the KIR allele diversity in pregnant women and investigate KIR and novel ligand binding in order to dissect the underlying mechanisms of KIR recognition with and without HLA-class I involvement in placental malaria infection. Further investigations are also warranted in other malaria endemic regions to assess the effects of racial differences, transmission intensities, and other environmental factors on the interaction between KIR genes and placental malaria.
